# INTERmittent FASTing in people with insulin‐treated type 2 diabetes mellitus – the INTERFAST‐2 study protocol

**DOI:** 10.1111/dme.14813

**Published:** 2022-02-28

**Authors:** Anna Obermayer, Norbert J. Tripolt, Peter N. Pferschy, Harald Kojzar, Angela Jacan, Markus Schauer, Faisal Aziz, Abderrahim Oulhaj, Felix Aberer, Caren Sourij, Barbara Obermayer‐Pietsch, Vanessa Stadlbauer, Harald Sourij

**Affiliations:** ^1^ Interdisciplinary Metabolic Medicine Trials Unit Division of Endocrinology and Diabetology Medical University of Graz Graz Austria; ^2^ CBmed – Center for Biomarker Research in Medicine Graz Austria; ^3^ Department of Epidemiology and Public Health College of Medicine and Health Sciences Khalifa University Abu Dhabi UAE; ^4^ Institute of Public Health College of Medicine and Health Sciences United Arab Emirates University Al Ain UAE; ^5^ Division of Cardiology Medical University of Graz Graz Austria; ^6^ Endocrinology Lab Platform Division of Endocrinology and Diabetology Medical University of Graz Graz Austria; ^7^ Division of Gastroenterology and Hepatology Medical University of Graz Graz Austria

**Keywords:** insulin sensitivity, insulin therapy, Intermittent fasting, type 2 diabetes mellitus (T2DM), weight loss

## Abstract

**Aim:**

Intermittent fasting, a dietary intervention of alternate eating and fasting, has gained popularity in people trying to lose weight. Intermittent fasting could provide an alternative to classic caloric restriction in people with type 2 diabetes mellitus. The aim of the study is to determine the impact of a 12‐week intermittent fasting regimen compared with usual care in people with type 2 diabetes mellitus receiving insulin therapy.

**Methods:**

This open, single‐centre, randomized controlled trial investigates participants with type 2 diabetes mellitus on insulin therapy and a glycated haemoglobin A1c (HbA1c) of ≥53 mmol/mol (≥7.0%) and a minimum insulin dose of 0.3 IU/kg body weight per day. Participants are randomized in a 1:1 ratio to either 12 weeks of intermittent fasting or the standard care group. All participants receive dietary counselling, continuous glucose monitoring, measurement of the resting metabolic rate, an oral glucose tolerance test, body composition measurement via dual‐energy X‐ray absorptiometry and stool samples for microbiome analyses at the beginning and at the end of the intervention. Two co‐primary outcomes (analysed in hierarchical order) were chosen for the study: (i) the difference in the change of HbA1c from baseline to 12 weeks and (ii) the difference in the number of participants achieving a combined end point encompassing a body weight reduction of at least 2%, an insulin dose reduction of at least 10% and an absolute HbA1c reduction of at least 3 mmol/mol (0.3%) between the two groups.

**What’s new**

- Blood sugar control and weight management can be challenging for people with type 2 diabetes mellitus.
- Intermittent fasting has become a popular option to manage weight, improve fasting glucose and insulin sensitivity.
- This study will investigate the feasibility and efficacy of intermittent fasting in people with insulin‐treated type 2 diabetes mellitus.
- The information gained will enhance our understanding of fasting interventions, which can be used to improve clinical dietary recommendations.

## 1 INTRODUCTION

Diabetes mellitus is characterized by chronic hyperglycaemia and impaired carbohydrate, lipid and protein metabolism caused by complete or partial insufficiency of insulin secretion and/or insulin action.^1^

Reduction of body weight has been a key strategy in mitigating the risk of developing diabetes.^2^ Among obese and people with prediabetes, a weight loss of 5%–7% improves fasting glucose and insulin sensitivity.^3^, ^4^ The most commonly used approach for weight loss is daily caloric restriction. In recent years, intermittent fasting has become a prominent approach because it consists of only a restricted food intake on either specific days of the week or during a predefined period of the day.

A subtype of intermittent fasting, alternate day fasting comprises food restriction and normal food consumption on a day‐to‐day alternating basis, has been shown to reduce body weight by 8% in obese people after 3–24 weeks of treatment. Additionally, a reduction in triglycerides, low‐density lipoprotein (LDL) cholesterol, systolic blood pressure and insulin resistance has been documented.^5^, ^6^, ^7^, ^8^

When comparing caloric restriction to intermittent fasting dietary regimens in humans, both dietary regimens achieve reductions in visceral fat mass, fasting insulin and insulin resistance in overweight and obese people.^9^

So far, available human data of intermittent fasting focusing specifically on people with type 2 diabetes mellitus treated with insulin, looking into potential glycaemic, body weight and treatment improvements, are mainly limited to case series.^10^, ^11^

Moreover, fasting and feeding schedule may interfere with the circadian rhythm and hence sleep length and quality^12^ and in addition, poor sleep has been associated with insufficient glycaemic control.^13^ The majority of the available data on intermittent fasting and sleep derives from Ramadan studies and were inconclusive with regard to sleep duration and sleepiness of people.^14^

People with type 2 diabetes mellitus have a distinct gut microbiome and disturbed diversity compared with healthy controls, shown to be able to affect and modulate lipogenesis, fat storage and metabolism^15^, ^16^, ^17^, ^18^, ^19^, ^20^ While dietary interventions are supposed to affect microbiome composition, research data on the effects of intermittent fasting on the microbiome in people with type 2 diabetes mellitus and insulin therapy is scarce and required.^21^, ^22^

The aim of the current study is to investigate safety and efficacy of intermittent fasting in people with type 2 diabetes mellitus and insulin therapy compared with usual care.

## 2 MATERIALS AND METHODS

### 2.1 Study design

This was an open, single‐centre, parallel, two‐arm, randomized controlled trial to evaluate the effect of intermittent fasting on participants with insulin‐treated type 2 diabetes mellitus over a period of 12 weeks. The investigations were performed at the Medical University of Graz, Division of Endocrinology and Diabetology, Interdisciplinary Metabolic Medicine Trials Unit. The protocol was approved by the Ethics Committee of the Medical University of Graz (EK 30‐350 ex 17/18). All participants gave written consent prior to any study‐related procedure. This study was conducted according to the principles of the Declaration of Helsinki, GCP‐ICH and the protocol and the requirements of the concerned regulatory authorities.

### 2.2 Inclusion and exclusion criteria

The study population consists of participants with type 2 diabetes mellitus, aged between 18 and 75 years (both inclusive) and a glycated haemoglobin A1c of ≥7.0% (≥53 mmol/mol). Main inclusion criteria: total daily insulin dose ≥0.3 units per kilogram of body weight and stable bodyweight in the preceding 3 months (change in weight <±3 kg). Participants who are willing to comply with study procedures, attend the study site, participate in the necessary protocols and comply with fasting protocols are included in the study. Main exclusion criteria contain active, known malignancies within the last year (excluding intraepithelial neoplasia of prostate, gastrointestinal tract and basalioma), pregnancy or intention of becoming pregnant, breastfeeding, a history of any chronic disease process that could interfere with interpretation of study results, new hormonal supplementation or contraceptive hormonal medication changes in the last 2 months, type 1 diabetes mellitus or other forms of diabetes mellitus, an acute or chronic inflammatory disorder, alcohol abuse with more than 15 standard drinks per week, overnight shifts or intake of illicit substances.

### 2.3 Sample size estimation

Sample size was estimated using the parameters of a previously published randomized controlled trial^23^ that investigated the effects of intermittent fasting on glycaemic control in people with type 2 diabetes mellitus. We based our sample size estimation on the baseline HbA1c data of this study 66 ± 7 mmol/mol (8.2 ± 0.6%) and estimated that a sample size of 23 participants in each group will have a power of 85% to detect a clinically important mean difference of 6 ± 7 mmol/mol (0.5 ± 0.6%) in HbA1c between the groups at a two‐sided alpha error of 5%. For the co‐primary outcome a sample size of 20 participants in each group would provide a power of 0.80 to detect a difference in proportion of 40% between the intermittent fasting and the control group. This calculation assumes 10% of the control group will reach the co‐primary end point (combined end point encompassing a body weight reduction of at least 2%, an insulin dose reduction of at least 10% and an absolute HbA1c reduction of at least 3 mmol/mol [0.3%]).

### 2.4 Recruitment of participants

Participants with insulin‐resistant type 2 diabetes mellitus are recruited from the outpatient clinics of the division of Endocrinology and Diabetology of the University Hospital Graz and form the Graz Diabetes Registry for Biomarker Research as well as via advertisement in local newspapers.

### 2.5 Randomization

Participants are randomly assigned to one of the two groups, the intermittent fasting group or the control group in a 1:1 ratio. Randomization is performed using the randomizer tool ‘Randomizer for Clinical Trials’: http://www.randomizer.at/ (accessed 21 Oct 2019) from the Institute of Medical Informatics, Statistics and Documentation of the Medical University of Graz, Austria.

### 2.6 Study hypothesis

We hypothesise that intermittent fasting over a period of 12 weeks can improve glycaemic control and body weight while being safe in people with type 2 diabetes and insulin therapy compared with usual care.

### 2.7 Study outcomes

#### 2.7.1 Primary outcomes

‐ The impact of intermittent fasting on the glucose metabolism in participants with insulin‐treated type 2 diabetes mellitus assessed by the difference in the change of HbA1c from baseline to 12 weeks.
‐ The difference in the number of participants achieving a combined end point encompassing a body weight reduction of at least 2%, an insulin dose reduction of at least 10% and an absolute HbA1c reduction of at least 3 mmol/mol (0.3%).

#### 2.7.2 Secondary outcomes

- Differences in the change
  - in resting metabolic rate (RMR) from baseline to 12 weeks
  - of glucose (area under the curve) within oral glucose tolerance test (oGTT) from baseline to 12 weeks
  - in glycaemic pattern (continuous glucose monitoring [CGM]) from baseline to 12 weeks
  - in body weight and body composition from baseline to 12 weeks
  - in body composition (fat mass/lean body mass) from baseline to 12 weeks
  - in mean 24 h blood pressure from baseline to 12 weeks
  - in microbiome composition from baseline to 12 weeks
  - in insulin dose from baseline to 4 weeks
  - in insulin dose from baseline to 8 weeks
  - in insulin dose from baseline to 12 weeks
  - on quality of life from baseline to 12 weeks
  - in the sleep quality and sleep disturbances from baseline to 12 weeks
  - in the sleepiness from baseline to 12 weeks
  - in physical activity from baseline to 12 weeks
- Differences in
  - time spent in hypoglycaemia, hyperglycaemia and number of hypoglycaemic events with symptoms and blood glucose <3 mmol/l (<54 mg/dl)
  - number of severe hypoglycaemic events from baseline to 12 weeks
  - of body weight after 64 weeks
  - of HbA1c after 64 weeks

#### 2.7.3 Justification of two primary outcomes

Lifestyle intervention trials in people with type 2 diabetes always face the problem that metabolic parameters, weight, and treatment can hardly be evaluated separately as they influence each other. For example, insulin dose decreases following weight loss will diminish the full impact of the weight loss on HbA1c reduction, while in contrast HbA1c reductions can simply be achieved by insulin dose increases without a contribution of the dietary intervention. Hence, as the parameters are interrelated, we believe that besides looking at the HbA1c change, it is also crucial to investigate a composite of weight, glycaemic control and insulin dose reduction. A weight loss of equal or more than 2% would indicate a weight change beyond the daily fluctuation.^24^ An insulin dose reduction of 10% or more is a pragmatic estimate and indicates better insulin sensitivity^25^ and an HbA1c reduction of at least 0.3% would be considered clinically meaningful.^26^

### 2.8 Intervention and investigations

**FIGURE 1:**
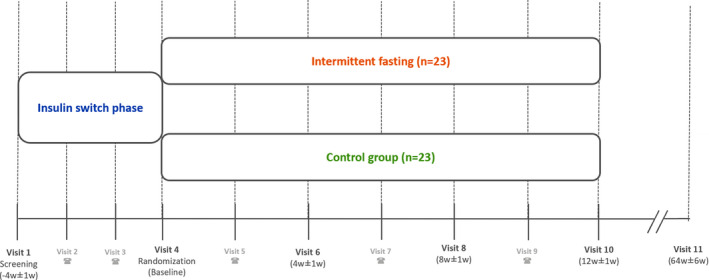
Flow chart of the intervention (an optional single follow‐up after 1 year will be added)

The flow chart of the intervention is shown in Figure 1.

46 participants (23 per group) with insulin‐treated type 2 diabetes mellitus are randomly assigned to ‘intermittent fasting’ or ‘control group’, in a 1:1 ratio. Intermittent fasting comprises 3 days (Monday, Wednesday, Friday) of 75% caloric restriction (i.e. only 25% of the recommended calorie intake; this intake is only allowed as breakfast and/or lunch to maintain an 18 hours period of fasting) and 4 days (Tuesday, Thursday, Saturday and Sunday) of 0% caloric restriction (i.e. ad libitum calorie intake) in 1 week.

#### 2.8.1 Biobank sampling

Serum and plasma samples are pseudonymized and stored at −80°C in the Biobank of the Medical University of Graz for potential future analyses.

#### 2.8.2 Laboratory measurements

Blood samples are obtained at all on‐site visits after a minimum of 8 h of overnight fasting (but should always take place after a normal feed day to ensure result comparability between groups) and processed by the local laboratory using standard methods for routine tests.

Insulin and c‐peptide are measured by chemiluminescence on an ADVIA Centaur system (Siemens Healthcare Diagnostics). Leptin is measured using an ELISA kit (DRG Instruments GmbH). Routine parameters are determined using a cobas^®^ analyzer (Roche Diagnostics).

#### 2.8.3 Peripheral blood mononuclear cell analysis

In a subgroup of up to 20 participants, 32 ml of peripheral blood is collected at baseline visit and visit 10 using BD Vacutainer CPT for the isolation of peripheral blood mononuclear cells. Peripheral blood mononuclear cells (PBMCs) will be further analysed using single‐cell sequencing technique in collaboration with the Division of Cardiology at the Medical University of Graz, Austria.

#### 2.8.4 Oral glucose tolerance test

Participants are asked to postpone their insulin injection until the end of the oGTT. Insulin, C‐peptide and glucose are measured before (−5), 15, 30, 60 and 120 min after ingestion of 75 g glucose (^®^Glucoral 75 citron, Germania Pharmazeutika). In addition, lipid parameters (total cholesterol, triglycerides, HDL, LDL, free fatty acids) and a routine safety lab are measured. The oGTT is performed after an overnight fast (apart from water) following an eating day (for the intermittent fasting group) and is evaluated by determining the area under the glucose curve.^27^

#### 2.8.5 Continuous glucose monitoring

Minimally invasive CGM measurement is performed using the Abbott FreeStyle Libre glucose monitoring device (Abbott Diabetes care). A sensor is introduced through the skin into the subcutaneous fat tissue. The upper arm is the main body location to applicate the sensors which are worn for 16 weeks (4 weeks insulin switch phase and 12 weeks intervention phase) by the participants. The Abbott sensor is factory calibrated and therefore does not require calibration by capillary prick blood. However, participants are instructed to scan the sensor with the corresponding recorder at least every 8 h. CGM data will be collected at on‐site visits.

#### 2.8.6 Non‐invasive 24h ambulatory blood pressure monitoring

Participants wear the blood pressure device for a 24‐h period. During this time, the device is programmed to inflate and record blood pressure at pre‐specified intervals (every 15 min during daytime hours and every 30 min during night‐time hours), which provides approximately 50–75 blood pressure recordings during the 24‐h period.

#### 2.8.7 Bone densitometry and body composition

Bone density scanning by dual‐energy X‐ray absorptiometry (DXA) is assessed with the GE Lunar iDEXA (GE Healthcare), estimating simultaneously body composition, for example, fat percentage according to the departmental Standard Operating Procedure. Body regions are defined using standard anatomical partitions. Scan areas are analysed to determine lean mass, fat mass, bone mineral density and total body composition.

#### 2.8.8 Bioelectrical impedance analysis

The BIACORPUS RX 4000 (Medical Healthcare GmbH) is used to assess body composition. It is a non‐invasive way to examine total body water and body composition by using eight adhesive electrodes and a frequency of 50 kHz.

#### 2.8.9 Activity measurement

The MoviSens‐device (Movisens GmbH) consists of a 5.3 × 3 × 2‐cm‐sized body and is fixed with a clip to the right hip. The three‐axial acceleration sensor has a range of ±8 g, a resolution of 12 bit and a sampling rate of 128 Hz. The recognition of different activities is based on the extraction of mathematical and statistical features of the raw acceleration signal. Calculated features are maximum frequency, step count, and the number of mean crossings.

#### 2.8.10 Faeces sampling and microbiome analysis

Sampling is performed with two stool collection tubes (Sarstedt), using a stool collector (Süsse Stuhlfänger). Directions for safe and hygienic faecal collection are provided to the participants of the study in words and in pictograms.

Bacterial DNA is extracted from stool samples using the MagNA Pure LC DNA Isolation Kit III (Roche). 16S rRNA is sequenced with next‐generation sequencing technology (Illumina MiSeq) and interpreted by the respective software analysers.

#### 2.8.11 Resting metabolic rate

The RMR is a component of energy expenditure that is measured by indirect calorimetry (IC). Participants must rest at least 30‐min after at least 8 h of sleep and after at least 3 h of fasting. The measurements take place for at least 30 min and are performed in standard neutral hospital room temperature. A breath mask is placed over the head of the participant. Oxygen consumption and carbon dioxide production are measured and energy expenditure is calculated by the Weir formula.^28^

#### 2.8.12 Sleep (sleep quality and sleep disturbances)

The Pittsburgh Sleep Quality Index (PSQI)^29^ measures self‐reported sleep quality and disturbances during the previous four weeks. PSQI has 19 items and measures 7 components of sleep: subjective sleep quality, sleep latency, sleep duration, sleep disturbance, use of sleeping medication, habitual sleep efficiency and daytime dysfunction.

#### 2.8.13 Sleep (sleepiness)

The Epworth Sleepiness Scale (ESS)^30^ is a self‐reported questionnaire that measures daytime sleepiness. The questionnaire consists of eight items with a respondents format 0 = would never doze, 1 = slight chance of dozing, 2 = moderate chance of dozing and 3 = high chance of dozing.

#### 2.8.14 Health‐related quality of Life

The EuroQol Questionnaire (EQ‐5D) measures health‐related quality of life. The questionnaire contains five dimensions: mobility, self care, usual activities, pain/discomfort and depression/anxiety.^31^

#### 2.8.15 International Physical Activity Questionnaire

The International Physical Activity Questionnaire will be used to measure health‐related physical activity.^32^

#### 2.8.16 Psychosomatic Competence Inventory

A questionnaire concerning interceptive awareness and self‐regulatory behaviour in relation to psychosomatic competence.^33^

#### 2.8.17 Dietary counselling

Dietary counselling is performed by a professional dietician and general recommendations are given to all study participants according to the recommendations of the German Nutrition Society (DGE) and personal caloric goals based on age and sex are discussed. At baseline, all participants receive one hour of general dietary counselling by a trained dietician. Participants are encouraged to consume a diverse and balanced diet, with three portions of vegetables and two portions of fruits per day (in general and on eating days). There is no restriction on macronutrient intake, but recommendations include the limitation of sugar and salt as well as the encouragement to consume more whole grains, legumes and smaller amounts of meat, dairy and fat. Water and unsweetened coffee and/or tea are suggested as beverages. Simmering food with small amounts of water and fat is encouraged over fried food. Participants are advised to take their time and to slowly eat meals to enjoy their food and encourage satiety. Though all participants have already received nutritional counselling years prior to the study when they initially started insulin therapy, the concept of prandial insulin dose estimation according to the carbohydrate amount eaten is repeated. All participants will have the same number of contacts with the dietician at the on‐site visits and phone visits for questions. 24‐hour emergency support is available to ensure the participants safety.

The participants of the intermittent fasting‐arm are given personal caloric goals for the fasting and eating days. The participants are presented with a couple of sample meals with a maximum of 500 kcal for the fasting days as a support to not exceed the recommended calorie maximum on those days.

#### 2.8.18 Insulin adaptation

To prevent hypoglycaemia caused by intermittent fasting,^23^ a specific protocol for insulin dose adaptation is used, adapted from Unnikrishnan et al.^34^ 4 weeks prior to the initiation of dietary intervention, participants switch to the same insulin treatment, consisting of glargine U300 as basal insulin and a short‐acting insulin. Insulin U300 is administered in the morning. Participants using premixed insulin receive 70% of their daily insulin dose as glargine U300 whereas participants using basal‐bolus insulin treatment continue on their original basal insulin dose but are switched to glargine U300. Participants who are allocated to the control group continue glargine U300 and prandial insulin as started in the switch phase with dose adjustments when deemed necessary by the treating physician.

Participants in the intermittent fasting group continue the same treatment as during the switch phase on the eating days and reduce the basal insulin by 20% on the fasting days with no prandial insulin. In case the blood sugar falls below 3.9 mmol/L, the fast is broken, and the glargine U300 dose is reduced by another 10% (on fasting and eating days) for the remainder of the study. (Table 1) Within the insulin switch phase, we standardise the insulin regimen but aim to sustain glucose control from screening without significant intensification of glucose control. During the intervention phase on days with 0% calorie restriction, we aim for 5.6–7.2 mmol/L in the fasting state and <11.1 mmol/L2h postprandial (for detailed insulin dose adjustments, see Table 2). Participants already using glargine U300 are able to start the intervention 7 days ±1 after the screening visit if treatment is stable at the discretion of the treating physician.

**TABLE 1:** Insulin regimen

	Eating days	Fasting days
Control group	Continue insulin dose from switch phase	Not applicable
Intermittent fasting group	Use insulin dose from switch phase	20% basal insulin reduction, no prandial insulin

**TABLE 2:** Insulin dose adjustment on fasting days and eating days

Fasting days	
<3.9 mmol/L	Fast stopped and additional 10% reduction of glargine U300 dose (on fasting and eating days), intake of 24 g of carbohydrates
3.9–5.5 mmol/L	Regular monitoring of glucose level trend
5.6–7.2 mmol/L	Target range
7.3–13.9 mmol/L	Regular monitoring of glucose level trend
>13.9 mmol/L	4 units of rapid acting insulin bolus and contact site for further insulin adjustment
Eating days	
Pre‐prandial 5.6–7.2 mmol/L	Target range – no insulin dose change required
Postprandial (2h) <11.1 mmol/L	Target glucose – increase prandial insulin by 2 IE if postprandial glucose is >11.1 mmol/l

#### 2.8.19 Statistical assessment

We defined two primary outcomes for the study to capture both changes in glucose control and the change of glucose control in conjunction with the insulin dose and the body weight changes. We will analyse these two primary end points in a hierarchical order, where the difference in the change of HbA1c from baseline to week 12 between the study groups is analysed first (at a significance level of 0.05). If the null hypothesis of this primary outcome (i.e. there is no difference in the change of HbA1c from baseline to week 12 between the two study groups) is rejected, the second primary outcome (i.e. the difference in the number of participants achieving a combined end point encompassing a body weight reduction of at least 2%, an insulin dose reduction of at least 10% and an absolute HbA1c reduction of at least 3 mmol/mol [0.3%]) will be tested at a significance level of 0.05. As the analysis will be performed in hierarchical order, according to the European Medical Agency (EMA) guideline on multiplicity issues in clinical trials (section 5.1.2.) no reduction or splitting of the α level is required.^35^

For the primary analysis of the primary outcomes, we will perform an intention‐to‐treat (ITT) analysis of all participants randomized using an unpaired t‐test for the change in HbA1c (alpha = 5%), followed by a chi‐square test comparing the two proportions of the co‐primary outcome (alpha = 5%), if the first test rejects the null hypothesis.

We will then perform sensitivity analyses for the primary outcomes using multivariate imputation by chained equations) for missing data in the ITT cohort. Additional sensitivity analyses will be performed in the per protocol cohort (people adherent to the intervention according to section 2.8.19) without and with data imputation for missing data.

Furthermore, analysis of covariance or multiple linear regression model adjusting for baseline HbA1c and multiple logistic regression model adjusting for baseline co‐primary, respectively, will be performed using the ITT population.

Finally, we will perform linear mixed‐effect models without multiplicity adjustment and generalized linear mixed models, respectively, for the primary outcomes in the ITT population.

Continuous secondary outcomes will be analysed using both unpaired t‐test and linear mixed‐effect models.

Transformation will be considered if data are not normally distributed. For categorical data, summary tables will include frequencies and percentages. For continuous data, means, standard deviations, medians and upper and lower quartiles will be presented. Change in outcomes over time, overall and by treatment groups will be displayed in line plots/boxplots with corresponding global and pairwise p‐values. All analyses will be performed in R version 4.1.0.

#### 2.8.20 Adherence to the protocol

Adherence to the protocol is assessed every 2 weeks with telephone calls and by discussing the food diary at the on‐site visits in all study participants. Furthermore, fasting periods can be assessed by the CGM. Adherence is defined as following the caloric restrictions (i.e. at maximum 25% of recommended calorie intake on a fasting day) on more than 75% of the fasting days.

#### 2.8.21 Mitigation of bias

Participants will be randomly assigned either to intermittent fasting group or control group in a 1:1 ratio. Randomization will be performed at the Institute of Medical Informatics, Statistics, and Documentation of Medical University of Graz, Austria. To include a population representative, we will recruit participants from the diabetes outpatient clinics of the University hospital Graz, Austria, the Graz Diabetes Registry for Biomarker research and via advertisements. This will ensure not to include people treated at a tertiary centre in Austria only.

#### 2.8.22 Gender‐related aspects

Data indicate that after 36 hours of fasting under the alternate day fasting dietary regimen (24 h total fast and 24 h ad libitum food consumption), men show an increased insulin sensitivity, whilst women show decreased insulin sensitivity.^36^ We will consider potential gender aspect within the study and therefore we aim to recruit participants at an approximate 50:50 gender ratio.

#### 2.8.23 Study status

First participant visit was 16 September 2019. As of 22 December 2021, we have recruited 46 participants (100%).

#### 2.8.24 Study registration

The study was registered at DRKS (Deutsches Register Klinischer Studien – German Clinical Trial Register) on 03.09.2019 as DRKS00018070.

## 3 STRENGTHS AND LIMITATIONS

One strength of this study is the continuous monitoring of the participants’ glucose levels with CGM to minimise the risk of hypoglycaemia. Another strength of this study is the insulin dose adjustment plan for fasting participants, which can be adapted and used by the participants beyond the scope of the study.

A challenge of this study is the fact the HbA1c level cannot be evaluated separately from the insulin dose. Hence, we have introduced a composite end point that captures glucose lowering, insulin dose reduction and body weight reduction. Another limitation is the duration of 12 weeks designed to establish the safety and efficacy of the intermittent fasting intervention in this participant group, however, further research is needed to explore the effects of intermittent fasting over a longer time period. Another limitation of this study is that the CGM is not blinded. Participants in the control group are able to see the changes in glucose levels depending on their food intake, which could lead to a change in eating habits potentially lowering HbA1c levels.

## 4 DISCUSSION

Previously, intermittent fasting was shown to cause weight loss of 3%–8% over 3–24 weeks and to reduce waist circumference by 4%–7%, which indicates that people lost some abdominal fat, associated with insulin resistance and related diseases.^37^ Although reducing insulin resistance is beneficial in people with diabetes, it also bears the risk of hypoglycaemia in people treated with insulin. Data from Ramadan studies are available, showing mitigation of hypoglycaemia during Ramadan using the flash glucose monitoring system.^38^ However, data from randomized controlled trials are largely missing.

Hence, our study aims to investigate the impact of a 12 weeks intermittent fasting intervention on glycaemic control, weight loss, insulin dose reduction and hence a combination of those single end point parameters. Moreover, this study will provide important information regarding the safety of intermittent fasting in people with type 2 diabetes mellitus using basal bolus insulin treatment and guidance on how the insulin dose should be adjusted in this population.

## CONFLICTS OF INTEREST

The authors declare that they have no competing interests.

## AUTHORSHIP

All named authors meet the International Committee of Medical Journal Editors (ICMJE) criteria for authorship for this article, take responsibility for the integrity of the work as a whole and have given their approval for this version to be published.

## AUTHOR CONTRIBUTIONS

HS conceived the trial. HS, NJT and AJ significantly contributed to development of the study protocol. NJT and AO contributed to efficient project management and day‐to‐day operation. HS and NJT contributed to acquiring ethical approval for the trial. AO, PNP, NJP, HK, BOP, VS, FAb, FAz and CS contribute to collection, analysis and interpretation of data. AOu contributed to the statistical analysis. MS is the dietician of the trial. AO wrote the final manuscript. All authors reviewed and contributed to the final manuscript.

## COMPLIANCE WITH ETHICS GUIDELINES

All procedures performed in studies involving human participants are in accordance with the ethical standards of the institutional and national research committee and with the 1964 Helsinki declaration and its later amendments or comparable ethical standards. Informed consent was obtained from all individual participants included in the study.

## Supporting information

Table S1
