# Supplementary material for: INTERmittent FASTing in people with insulin‐treated type 2 diabetes mellitus – the INTERFAST‐2 study protocol
Source: Diabet Med. 2022 Feb 28;39(6):e14813. doi: 10.1111/dme.14813 (PMC9304224; doi:10.1111/dme.14813)
Supplement: Supplementary file 1 — Table S1 [file DME-39-0-s001.docx]

| **Task** | **Visit** | | | | | | | | | | |
| --- | --- | --- | --- | --- | --- | --- | --- | --- | --- | --- | --- |
|  | **1**  **on site**  ***-4w±1w*** | **2**  **phone**  ***-18d±3d*** | **3**  **phone**  ***-9d±3d*** | **4**  **on site**  ***Baseline*** | **5**  **phone**  ***2w±3d*** | **6**  **on site**  ***4w±1w*** | **7**  **phone**  ***6w±3d*** | **8**  **on site**  ***8w±1w*** | **9**  **phone**  ***10w±3d*** | **10**  **on site**  ***12w±1w*** | **11**  **On site**  **64w*±6w*** |
| Informed Consent | X |  |  |  |  |  |  |  |  |  |  |
| In- /exclusion criteria | X |  |  |  |  |  |  |  |  |  |  |
| Randomization |  |  |  | X |  |  |  |  |  |  |  |
| Participant brochure | X |  |  |  |  |  |  |  |  |  |  |
| Demography, medical history | X |  |  | X |  |  |  |  |  |  | X |
| Concomitant medications | X |  |  | X |  | X |  | X |  | X | X |
| Physical examination | X |  |  | X |  | X |  | X |  | X | X |
| Blood sampling | X |  |  | X |  | X |  | X |  | X | X |
| Non-invasive 24h ambulatory blood pressure monitoring |  |  |  | X |  |  |  |  |  | X |  |
| Bone densitometry and body composition |  |  |  | X |  |  |  |  |  | X |  |
| Insulin switch to IGlar U300 | X |  |  |  |  |  |  |  |  |  |  |
| Insulin dose adaptation |  | X | X | X | X | X | X | X | X |  |  |
| Dietary counselling |  |  |  | X |  | X |  | X |  |  |  |
| Feces sampling |  |  |  | X |  | X |  |  |  | X |  |
| Resting metabolic rate |  |  |  | X |  |  |  |  |  | X |  |
| Activity measurement | X |  |  | X |  | X |  | X |  | X |  |
| Continuous glucose monitoring (CGM) | X | X | X | X | X | X | X | X | X | X |  |
| Oral glucose tolerance test (OGTT) |  |  |  | X |  |  |  |  |  | X |  |
| Pregnancy test | X |  |  | X |  | X |  | X |  | X |  |
| International Physical Activity Questionnaire (IPAQ) | X |  |  | X |  | X |  | X |  | X |  |
| Sleep questionnaires |  |  |  | X |  | X |  | X |  | X |  |
| Health Related Quality of Life |  |  |  | X |  | X |  | X |  | X |  |
| AE/SAE assessment |  |  |  | X |  | X |  | X |  | X |  |
| Bioelectrical impedance analysis (BIA) |  |  |  | X |  | X |  | X |  | X |  |
| Psychosomatic Competence Inventory (PSCI) |  |  |  | X |  |  |  |  |  | X |  |
